# Revisiting the Marrow Metabolic Changes after Chemotherapy in Lymphoma: A Step towards Personalized Care

**DOI:** 10.1155/2011/942063

**Published:** 2011-09-28

**Authors:** Bingfeng Tang, Malaykumar M. Patel, Regina H. Wong, Daniel Wood, Christiana O. Wong, Dafang Wu, Pek Lan Khong, Ching Yee Oliver Wong

**Affiliations:** ^1^Department of Radiology, The Ohio State University College of Medicine, Columbus, OH 43210, USA; ^2^Department of Radiology, Oakland University William Beaumont School of Medicine, Royal Oak, MI 48073, USA; ^3^LSA, The University of Michigan, Ann Arbor, MI 48109, USA; ^4^Saybrook College, Yale University, New Haven, CT 06511, USA; ^5^Department of Diagnostic Radiology, University of Hong Kong, Hong Kong, China; ^6^Positron Diagnostic Center and Medical Cyclotron, Department of Nuclear Medicine, William Beaumont Hospital, 3601 W, Thirteen Mile Road, Royal Oak, MI 48073, USA

## Abstract

*Purpose*. The aims were to correlate individual marrow metabolic changes after chemotherapy with bone marrow biopsy (BMBx) for its potential value of personalized care in lymphoma. *Methods*. 26 patients (mean age, 58 ± 15 y; 13 female, 13 male) with follicular lymphoma or diffuse large B-cell lymphoma, referred to FDG-PET/CT imaging, who had BMBx from unilateral or bilateral iliac crest(s) before chemotherapy, were studied retrospectively. The maximal standardized uptake value (SUV) was measured from BMBx site over the same area on both initial staging and first available restaging FDG-PET/CT scan. *Results*. 35 BMBx sites in 26 patients were evaluated. 12 of 35 sites were BMBx positive with interval decrease in SUV in 11 of 12 sites (92%). The remaining 23 of 35 sites were BMBx negative with interval increase in SUV in 21 of 23 sites (91%). The correlation between SUV change over the BMBx site before and after chemotherapy and BMBx result was significant (*P* < 0.0001). *Conclusions*. This preliminary result demonstrates a strong correlation between marrow metabolic changes (as determined by FDG PET) after chemotherapy and bone marrow involvement proven by biopsy. This may provide a retrospective means of personalized management of marrow involvement in deciding whether to deliver more extended therapy or closer followup of lymphoma patients.

## 1. Introduction

Molecular imaging using 2-deoxy-2-[F-18]fluoro-d-glucose (FDG) positron emission tomography (PET) scanning has recently emerged as a major imaging modality for the staging and followup of patients with non-Hodgkin's lymphoma (NHL) [1, 2]. Diffuse large B-cell lymphoma is the most common subtype. Follicular lymphoma accounts for 22% of NHL in adults with high tendency to involve the bone marrow [3, 4]. Follicular grade I lymphoma is the most predominating histological subtype to involve the marrow [5]. Before initiation of treatment, distinguishing potentially curable (stage I/II) from advanced disease (stage III/IV) may guide the planning of management. The advanced stages III and IV correlate significantly with shorter overall or event-free survival, and treatment may have to be modified accordingly. In NHL, bone marrow involvement places the patient in the advanced disease (stage IV). Bone marrow biopsy (BMBx) is the established method for detection of bone marrow involvement in the initial staging and restaging of NHL. However, BMBx is a painful and invasive procedure and sometimes only a small sample can be obtained, which may be inconclusive due to sampling errors, despite bilateral iliac crest blind biopsy under anesthesia. Furthermore, even if the volume of the biopsy is adequate, focal lesions may be missed. Thus, although it is very specific, BMBx from traditional biopsy sites (iliac crests) has low sensitivity in detecting marrow involvement of lymphoma. It is essential to have a supplementary prospective, and if not possible even retrospective diagnostic procedure, possibly consisting of a multistep approach, to reliably assess bone marrow infiltration in patients with NHL to compliment the BMBx.

The ability of FDG-PET to evaluate bone marrow infiltration in patients with lymphoma has been investigated extensively. Multiple prior studies showed that FDG-PET has a high potential to detect bone marrow involvement in high-grade malignant lymphoma with low sensitivity for the detection of bone marrow infiltration in low-grade NHLs [6, 7] despite the fact that bone marrow involvement occurs in 30%–50% of patients with NHL. The majority of these studies used visual interpretation of marrow FDG uptake during whole-body staging PET scans to assess bone marrow involvement. Since it is more common in indolent histology [8, 9], the marrow activity may be not avid for visual detection based on a single staging PET scan. In the visual approach, the marrow was assumed to be abnormal where the FDG uptake was equal to or greater than uptake in the liver, which was usually greater than background. This approach for assessing marrow involvement depends on the extent of marrow infiltration by lymphoma and has made no use of available information from pre- and postchemotherapy intramedullary metabolic activities due to changes in cell population.

The standardized uptake value (SUV) is a semiquantitative measure of the glucose metabolism based on the degree of FDG uptake, which is derived from the tumor activity divided by dose per body mass in the attenuation-corrected PET images [10]. It may improve the definition of abnormal areas by reducing subjective assessment. It is common to see the pattern of diffusely increased FDG uptake in normal bone marrow after chemotherapy on F-18 FDG-PET scans due to change in hematopoietic cell population. Decreased FDG uptake after chemotherapy is noted in the areas with PET evidence of bone marrow involvement due to reduction in tumor population. The aims were to correlate individual marrow metabolic changes after chemotherapy with bone marrow biopsy (BMBx) for its potential value of personalized care in lymphoma.

## 2. Materials and Methods

Database of patients referred for FDG-PET/CT scan for initial staging and first restaging after chemotherapy who had BMBx from unilateral or bilateral iliac crest(s) before chemotherapy at a tertiary hospital and cancer center over a period of two years was retrospectively searched with Human Investigation Committee approval. Patients were excluded if they had malignancies other than lymphoma or if they had received prior radiation treatment or chemotherapy. Twenty-six patients were eligible for this study. The mean age was 58 ± 15 years old with 13 females and 13 males. There were 16 follicular lymphomas (FL, grades I, II, and III) and 10 diffuse large B-cell lymphomas (DLBC). The maximal standardized uptake value (SUV) was measured from BMBx site over the same area on both initial staging and restaging FDG-PET/CT scans. The interval changes of SUV were classified as decrease or increase and correlated with BMBx result of positive or negative for bone marrow involvement by lymphoma. 

PET-CT imaging was obtained by a dedicated 16-slice body PET-CT scanner (GE Discovery DST, GE Medical Systems, Milwaukee, Wis, USA). All patients had fasted for 4 to 6 hours before the intravenous injections of average of 15 mCi (555 MBq) F-18 FDG. PET scans were performed around one and a half hours after injection right after mapping CT. This choice of FDG uptake time was based on the modified schedule of usual one-hour time of FDG uptake to maximize the contrast between tumor, soft tissue, and benign inflammation if any [11]. The PET images were obtained at each bed position for 3 minutes with 6–8 beds to cover the entire body. The PET images were obtained using a two-dimensional high-sensitivity mode with an axial field of view of 15 cm in a 256 × 256 matrix. A 3-slice overlap was utilized between the bed positions. The PET images were reconstructed iteratively on a 128 × 128 matrix using ordered-subsets expectation maximization algorithm for 30 subsets and two iterations, with a 7.0 mm postreconstruction filter. An initial scout scan was obtained first to define the imaging field for the CT component of image acquisition, which used the following imaging parameters: 140 kVp, 120–200 mA, 0.8 seconds per CT rotation, pitch 1.75 : 1, and detector configuration of 16 × 1.25 mm, 3 mm slice thickness with oral contrast only.

The serum glucose in mg/dL was recorded just before PET, and the maximum SUV, defined as tumor activity divided by dose injected per body mass, was measured by searching the maximum value within a volume of interest over BMBx sites (posterior iliac crests) by a nuclear medicine physician. Bone marrow involvement was defined by histopathologically proven bone marrow lymphoma infiltration from marrow biopsy. Statistical analysis was done using SPSS (SPSS Inc, Chicago, IL) for comparing the changes of marrow before and after chemotherapy. A *P* value of <0.05 was considered significant in all tests.

## 3. Results

**Figure 1:**
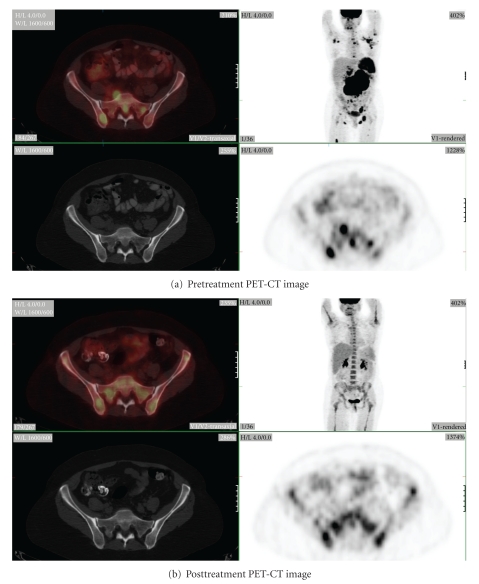
53-year-old female with follicular grade 3 lymphoma with clear marrow involvement (a). This patient had bilateral iliac marrow biopsy. The post-treatment PET-CT (b) shows diffuse uniform increase in marrow uptake. However, the SUV actually decreases (from 6.7 to 4.9) on the right iliac bone marrow with pathologic confirmation of marrow involvement (b). On the contrary, the SUV over the pathologically negative left iliac bone marrow increases (from 2.7 to 4.2) due to normal hematopoietic response.

Thirty-five BMBx sites from 26 patients were evaluated. Twelve of 35 sites were BMBx positive with interval decrease in SUV in eleven out of the 12 sites (92%) (Figure 1). For the 11 sites with true positive PET findings (decreased SUV on BMBx positive sites), the magnitude of SUV decrease ranged from 0.7 to 15.8 (−27 to −89%) for an average 8.3 ± 5.9 (−64 ± 24%).

**Figure 2:**
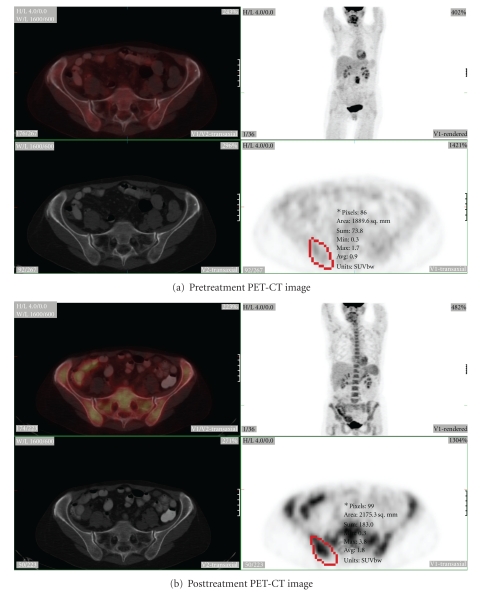
58-year-old female with follicular grade 3 lymphoma (a) without focal evidence of marrow involvement by PET. She had right iliac biopsy. The posttreatment PET-CT (b) again shows diffuse increase in marrow uptake. The SUV increases (from 1.7 to 3.8) on the right iliac bone marrow (red region) with pathologic examination negative for marrow involvement. The change of the SUV over bone marrow is all due to normal hematopoietic response to chemotherapy.

The remaining twenty-three of 35 sites were BMBx negative with interval increase in SUV in 21 of 23 sites (91%) (Figure 2). In the 21 sites with true negative PET results (increased SUV on BMBx negative sites), the range of SUV increase was from 0.1 to 9.2 (+5 to +575%), average 1.7 ± 2.0 (+106 ± 128%).

The correlation between SUV change over the BMBx site before and after chemotherapy and BMBx result was significant (*P* < 0.0001). The interval between chemotherapy and restaging PET scan was 9 to 75 days (mean 22 ± 16 days). The interval between BMBx and initial staging PET scan was 1 to 47 days (mean 8 ± 9 days).

## 4. Discussion

It is currently a common practice to use PET scan as a qualitative tool in the arena of lymphoma, supplemented by more clinically useful information extracted in the form of SUV, to aid in therapeutic decision making and prognostication. The clinical significance of the current study suggested that change in marrow SUV measurement after chemotherapy might provide a retrospective marrow staging, which may give the same information noninvasively as obtained from traditional BMBx regarding bone marrow involvement of lymphoma. 

Bone marrow involvement in patients with lymphoma signifies extensive disease with less favorable prognosis. Although bone marrow biopsy is still the gold standard method for detection of bone marrow involvement in the initial staging or restaging of lymphomas, the potentially low sensitivity and invasive nature as well as other limitations of marrow biopsy make it nonideal diagnostic test in detection of marrow infiltration. A more reliable and sensitive noninvasive method of detecting marrow involvement would be desirable. FDG-PET imaging has become a major imaging modality for the staging and followup of patients with NHL [1, 2], and the potential ability of FDG-PET to evaluate both focal and diffuse bone marrow infiltration in patients with lymphoma is a natural choice for investigation and optimization. Though prior studies with pure visual interpretation of marrow FDG uptake to assess marrow have revealed a high potential to detect bone marrow involvement in malignant lymphoma [12–14], the sensitivity is still unacceptably low in low-grade non-Hodgkin's lymphomas [6, 7, 15]. The present study was undertaken to investigate and optimize the efficacy of FDG-PET as a potentially improved and complimentary method to aid in evaluating marrow involvement in patients for personalized care of lymphomas by utilizing individual interval metabolic changes instead of detection of focal abnormal marrow uptake based on a single staging PET scan alone.

The strong correlation between SUV change after chemotherapy and BMBx result as demonstrated from this current study has notable potential clinical significance. PET-CT is more sensitive than marrow biopsy and can be employed routinely to assess the entire marrow [16]. In patients with initial obvious focal FDG-avid bone marrow lesions, FDG-PET may offer guidance to biopsy or can be used as direct evidence of bone marrow involvement. The bone marrow involvement of these lesions can be confirmed by analyzing SUV changes after chemotherapy. If initial FDG PET shows no definite focal marrow lesions and patient needs chemotherapy clinically, then the retrospective analysis of SUV changes after chemotherapy over the traditional BMBx sites may offer similar if not better information as obtained from BMBx regarding bone marrow involvement by lymphoma and therefore potentially eliminate the error on management caused by false negative pretreatment BMBx due to sampling issue. Whether a blind marrow biopsy may still be warranted in patients with indolent lymphoma for whom chemotherapy may not be offered if there is no evidence of bone marrow involvement by lymphoma, or in patients with marrow population changes related to factors other than chemotherapy only, for example, G-CSF treatment, bone marrow dysplasia, prior chemo- or radiation treatment, and so forth, needs further clinical investigation. Nonetheless, the current study moves a step towards personalized care by employing retrospective evidence of marrow involvement in lymphoma patients. Since the current revised response criteria for lymphoma require clearance of infiltrative marrow lesions by repeated biopsy [17], the current study may offer an alternative insight for noninvasive marrow response criteria by requiring involved marrow sites to show decrease in SUV from hot or normal FDG uptake to reduced or cold metabolic activity on the marrow. 

With PET-CT, the functional PET images are coregistered with the anatomic CT images obtained by the almost simultaneously acquired CT scan. This approach can result in a significant improvement in the accurate anatomic localization and the region of interest determination and therefore make sure that the measurement of SUV is over the same area before and after chemotherapy. The current study demonstrates that the change in metabolic behavior correlates well with marrow cell population change and suggests possible optimization of metabolic information by using intramedullary SUV change to countercheck or supplement the invasive limited sampling of histological examination to predict marrow involvement, which in turn may lead to the most appropriate subsequent management for each individual patient. 

 There are limitations of this current retrospective study, including small sample size, mixed histopathological classification, and significant variation of interval between chemotherapy and restaging PET scan. A comparison between bone marrow biopsy and evaluations of FDG-PET after treatment is not accomplished. This is partially because there will be little justification to repeat biopsy if there is already a good response to therapy. A well-designed prospective study overcoming the above limitations to further confirm the conclusion of this preliminary study is necessary and is currently being investigated.

## 5. Conclusion

This preliminary study demonstrates a strong correlation between marrow metabolic changes by FDG PET scan after chemotherapy and marrow cell population change. There is an inverse relationship between (positive) marrow involvement of lymphoma and SUV change (decrease) after chemotherapy. The potential clinical significance of this observation is to provide a noninvasive retrospective means of countercheck or replacement of the invasive limited sampling of histological examination in predicting marrow involvement, which in turn may help in personalized care of deciding whether to deliver more extended therapy or closer followup of lymphoma patients.
